# What can we learn from studies on regional differences in the utilization of laboratory tests?

**DOI:** 10.3109/03009734.2011.624281

**Published:** 2011-10-29

**Authors:** Anders Larsson

**Affiliations:** Department of Medical Sciences, Uppsala University

In this issue of *Upsala Journal of Medical Sciences* we publish an interesting and highly novel article on the use of laboratory tests in Spain (1).

The cost for health care has grown steadily over the last 50 years. The yearly growth has been higher than the growth of the gross domestic product (GDP). Such growth cannot continue indefinitely, and we have over the last two decades seen different ways of trying to limit the growth of health care costs. This has resulted in a slight shift in the aim of health care from the best care possible for the patient to the best care possible for the patient at a ‘reasonable price’.

Today, we want as much health care as possible for our citizens per spent euro, dollar, or whatever currency we are using. To be able to evaluate this, we need to study the benefits of different treatment strategies and compare them with others. Usually, this is analyzed in prospective studies that take many years before we have any answers, and it is rather costly. Another method could be to study regional differences in clinical praxis and compare them with outcome. An example of this is that the surgeon hears about a new surgical technique and if it is superior to the present one it is introduced at his/her hospital. Such method transfers usually depend on individuals and are not very systematic. In Sweden and in many other countries quality registers are developed to find regional differences as a way of improving health care. The disadvantage of the quality registers is that they are limited to a single country; it would be even more interesting to compare different countries as the differences are probably much larger between countries than within.

The present Spanish study gives valuable insights into the Spanish tradition of laboratory testing and allows comparisons with the praxis at our own hospitals (1). The cost of individual laboratory tests is usually low, which may have contributed to the limited number of studies on the utilization of tests in different countries. The role of laboratory tests has evolved greatly over time, and the number of tests has increased dramatically since the middle of the last century. Presently, laboratory tests are used in large numbers and are involved in 60%–70% of all critical decisions such as admittance, discharge, and medication (2).

Laboratory tests are ordered for many different reasons, and some of the most common ones are for definition of a diagnosis, assessment of disease severity, monitoring therapy, and detection of complications. It is important to remember that the goal of laboratory testing is not the acquisition of information per se but to improve the outcome for the patient. There is no clear correlation between the number of tests and the quality of care (3,4). Excess test ordering has been reported to represent as much as 30%–40% of all tests (5), and 20%–95% of selected tests (6). Thus, there are strong indications that laboratory tests are overused, but they may also be underused and misused. It is usually the most frequently ordered laboratory tests that are overused (7).

Spanish test-ordering patterns differ clearly from what could be considered a ‘normal’ Swedish pattern (8). It is easy to say that what we do in our own hospital is correct and everything else is wrong (regardless of whether we work in Spain or in Sweden). For instance, from a Swedish perspective the number of serum urate tests seems high in Spain, and also serum iron is usually ordered without an accompanying transferrin or total iron binding capacity (TIBC) test. The Swedish tradition is to limit urate tests mainly to patients with suspected gout and to combine iron with transferrin/TIBC to allow calculation of iron saturation. It seems unlikely that the number of gout patients is that much higher in Spain than in Sweden. Does this mean that Spanish tradition is wrong? Not necessarily, as it could mean that Spanish doctors use urate for other types of patients. For instance, Spanish doctors may be using it as a tumor marker. Urate increases with increased cell turnover, and it is often increased in cancer patients. It would be very interesting to know why Spanish doctors order more urate tests. Patients in Spain and Sweden should suffer from approximately the same types of diseases. The difference between the two countries shows that there is a suboptimal use of urate in at least one of the countries. It is thus worth exploring the use of urate in both countries. I still believe that iron should not be ordered without transferrin as in the Spanish study. Maybe I am wrong, but I would still not recommend a change in Swedish test use. Maybe both countries are wrong, and we should use ferritin or soluble transferrin receptor instead of iron, and transferrin when suspecting iron deficiency anemia. Both iron and iron saturation have a clear diurnal variation, which makes the interpretation of test results difficult if the patients come to the clinic at other times than in the morning.

It could be argued that it is difficult to define the optimal use of specific laboratory markers, but the differences shown in the Spanish study can be used efficiently for discussions with local doctors on the use of laboratory tests to change their ordering pattern. We reduced AST requests from primary care doctors significantly by discussing the clinical value of the marker. We could show that the hospital in Odense, Denmark, had reduced the number of AST tests to almost zero but there were no indications that patient care in Odense was worse than in our county. The conclusion from the comparison was that a lot of our AST tests were redundant. The discussion led to a significant decrease in AST requests. According to my experience, this type of discussion is much more efficient for changing ordering habits than using a higher price or other types of pressure to change ordering habits. Actually, we also tried doubling the AST price as a means to reduce the number of tests, but the effect of the cost increase was negligible. It seems as if force only results in short-term changes at best, and the request patterns usually return to their original state within a few months. If we want to create permanent changes we need continuous education on the use of laboratory tests. Studies on regional use of laboratory tests provide an excellent base for this type of education.

## References

[1] Salinas M, López-Garrigós M, Diaz J, Ortuño M, Yago M, Laíz B (2011). Regional variations in test requiring patterns of general practitioners in Spain. Ups J Med Sci.

[2] Forsman RW (1996). Why is the laboratory an afterthought for managed care organizations?. Clin Chem.

[3] Schroeder SA, Schliftman A, Piemme TE (1974). Variation among physicians in use of laboratory tests: relation to quality of care. Med Care.

[4] Daniels M, Schroeder SA (1977). Variation among physicians in use of laboratory tests. II. Relation to clinical productivity and outcomes of care. Med Care.

[5] Rao GG, Crook M, Tillyer ML (2003). Pathology tests: is the time for demand management ripe at last?. J Clin Pathol.

[6] Axt-Adam P, van der Wouden JC, van der Does E (1993). Influencing behavior of physicians ordering laboratory tests: a literature study. Med Care.

[7] McConnell TS, Berger PR, Dayton HH, Umland BE, Skipper BE (1982). Professional review of laboratory utilization. Hum Pathol.

[8] Mindemark M, Larsson A (2011). Longitudinal trends in laboratory test utilization at a large tertiary care university hospital in Sweden. Ups J Med Sci.

